# “With compliment from...”: A critical reflection on the function and use of compliment letters addressed to hospital staff

**DOI:** 10.1017/S1478951525101065

**Published:** 2025-11-07

**Authors:** Valérie Clerc, Friedrich Stiefel, Béatrice Schaad, Laurent Michaud, Céline Bourquin

**Affiliations:** 1 Psychiatric Liaison Service, Lausanne University Hospital (CHUV) and University of Lausanne, Lausanne, Switzerland; 2 Center for Patient, Family and Professional Experiences, Lausanne University Hospital (CHUV) and University of Lausanne, Lausanne, Switzerland

The experience of being a patient or the significant other (SO) of a patient prompts some individuals to provide written feedback to healthcare professionals and hospitals. Negative feedback is increasingly collected to improve quality of care through complaint centers and mediation processes (Reader et al. 2014; Schaad et al. 2019). In contrast, positive feedback has received little attention and is still rarely drawn upon in the healthcare setting (Gillespie and Reader 2021). Recently, authors have argued that it can be employed to identify aspects of care that matter to patients and families (Aparicio et al. 2017, 2019), improve the quality of care (Gillespie and Reader 2021; Herbland et al. 2017; Plunkett 2022), and ultimately drive change in healthcare (Lloyd et al. 2023). Compliment letters addressed to hospital staff represent a significant portion of this positive feedback. In the literature, compliment letters are most commonly regarded as expressions of satisfaction and gratitude (Aparicio et al. 2017; Gillespie and Reader 2021) and have been investigated for their impact on healthcare professionals, particularly in relation to increased motivation and reduced burn-out (Aparicio et al. 2019, 2022b; Herbland et al. 2017; Martins Pereira and Hernández-Marrero 2016; Stirling et al. 2023).

However, considering compliment letters solely as expressions of gratitude overlooks the fact that they also serve as testimonies of patients’ and significant others’ experiences with the healthcare system. These letters indeed convey a wealth of information (Herbland et al. 2017) and may have specific functions for their writers. A recent study^1^ we conducted in the neonatology unit of a university hospital showed that, in addition to praising the care their newborns received, parents recounted their experiences and feelings during the hospital stay, described their observations of healthcare professionals’ work and attitudes, and provided feedback on their children’s progress after discharge (Clerc et al. 2024). Compliment letters thus allowed parents not only to express their gratitude, but also to sustain their emotional recovery, facilitate a return to normality, and maintain a connection with healthcare providers. We therefore argue that this type of positive feedback is part of a sense-making process and should be understood as such.

In this reflection paper, informed by our study findings, we propose to examine and explore more deeply the issue of compliment letters from a psychological-interactional and systemic perspective.

## Beyond the manifest: The psychological and interactional dimension of compliment letters

Compliment letters convey the experiences of patients and their SO’ to healthcare professionals and are often driven by latent, non-conscious needs. They thus reveal both the writers’ subjectivity and the nature of their relationship with the recipients. These subjective and relational dimensions are certainly shaped by the medical situation, which is characterized by vulnerability and dependency on the part of hospital users, as well as by the moral contract assumed by healthcare providers, who usually merit the trust of the vulnerable. When this moral contract is perceived as being respected, positive experiences with feelings of gratitude may emerge; conversely, when the moral contract is broken, negative experiences accompanied by feelings of betrayal may result. However, compliment letters are also influenced by what has been conceptualized in psychoanalytic theory as transference, suggesting that such expressions of satisfaction are colored by past experiences of the writers with their significant relational figures (Balint 1955; Gabbard 2017). Positive transference can develop from past experiences of empathic attention and support, fostering feelings of gratitude or affection. It may also arise when current care is perceived as reparative in light of earlier unmet emotional needs. Negative transference, associated with emotions such as anger or anxiety, may, on the other hand, stem from conflictual relationships with prior authority figures or from insecure attachment during development, leading to increased sensitivity and a tendency to interpret unavoidable gaps between patients’ needs and their fulfillment as signs of neglect or even mistreatment. Transference reactions occur in everyday life but are especially prominent in the context of care and disease (Balint 1955), where relational asymmetry assigns healthcare professionals a parental role and activates past experiences of dependency. Positive and negative feedback must therefore be addressed by considering the transferential dynamics inherent in medical care.

From an interactional perspective, compliment letters may also be motivated by a desire for resonance with those who provided care, a way to maintain connection by bearing witness to past or present experiences or outcomes of care. Even if healthcare professionals do not respond, the fundamental human need to bear witness (Frank 1995), especially after traumatic experiences, serves as a means of relating, even if no echo is received (as with some prayers). It seems to us that both intrapsychic and interactional forces operate within these compliment letters.

Research on compliment letters (and other forms of positive feedback) from patients and SO, as well as their use by hospitals and in healthcare settings, should therefore take into account these transferential and interactional dimensions. They reflect an important aspect of medical care that is often relegated to the background during times of financial constraints, pressures on clinical productivity, and a focus on quantitatively measurable outcomes of medical treatments. Treatment and care are two distinct but equally necessary dimensions of medicine. Taking these latent dimensions into account can shed light on the psychological experience of being a patient or the SO of a patient as well as on the relational needs triggered by illness. This can help to orient medicine toward its core missions.

## Using and misusing compliment letters in hospital and healthcare settings

Studies have explored the specific aspects for which patients or SO expressed gratitude (Aparicio et al. 2017, 2019; Herbland et al. 2017), as well as the impact of gratitude on healthcare professionals (Aparicio et al. 2022a, 2019, 2022b; Stirling et al. 2023). This body of research suggests that expressions of gratitude from patients and SO can help reduce staff stress and burnout, while also enhancing motivation, job satisfaction, productivity, and overall well-being among healthcare professionals (Aparicio et al. 2022a, 2019, 2022b; Lloyd et al. 2023). Based on these findings, some authors have proposed that compliment letters could be used by healthcare institutions to foster these positive outcomes (Aparicio et al. 2022a, 2019, 2022b). Practical implementations include displaying compliment letters in shared spaces such as cafeterias, hallways, restrooms, or notice boards within hospitals or care units (Aparicio et al. 2019, 2022b; Herbland et al. 2017). This proposition is part of a broader strategy that encourages managers and key stakeholders to actively support and facilitate the collection and dissemination of patients’ expressions of gratitude (Lloyd et al. 2023).

We believe it is important to critically examine the use of compliment letters as a means of enhancing healthcare providers’ well-being and performance. Such a utilitarian approach risks overlooking the writers’ subjectivities, as well as their intrapsychic and interactional expressions and needs, thereby reducing compliment letters to a mere means to an end. This utilization also shifts compliment letters from being personal messages from patients and SO to institutional messages directed at healthcare providers, conveying entirely different meanings and goals. Moreover, a lack of recognition from patients and SO (in contrast to receiving compliment letters) would therefore be falsely linked to the quality of care provided by healthcare professionals. However, healthcare professionals are themselves subject to contextual factors such as time constraints, administrative burden, and productivity pressure, which help explain this outcome and affect them as much as they affect the patients and SO (Bourquin et al. 2022). Initiatives that rely on the gratitude of patients and SO, such as through compliment letters, may thus implicitly convey the message to healthcare professionals: “Do better without supplementary resources”. Real and negative conditions within the institution are deflected by an emphasis on the positive.

## Compliment letters and the implementation of the biopsychosocial model

If the handling of compliment letters is managed like a means to intervene on healthcare professionals to enhance institutional gains, it neglects the fact that these letters are expressions of patients’ and SO’ experiences and needs. These experiences and needs arise from the clinical encounter and are shaped by conscious and non-conscious intrapsychic and interactional dynamics. Such handling contributes to the emergence of a system in which “patients are consumers; doctors are providers; healthcare is a commodity,” as Druvh Khullar explains in a recent article in The *New Yorker* (Khullar 2024).

Almost 50 years ago, George Engel called for a paradigm shift in medicine through the creation of the biopsychosocial model of disease (Engel 1977). Significant progress has been made in this direction, particularly in the care of patients with chronic diseases. Initiatives such as patient empowerment, the implementation of palliative care, and the development of consultation-liaison services reflect a growing awareness of the psychosocial dimensions of diseases. Yet the biopsychosocial model applies not only to diseases, patients, and clinical settings but also to healthcare professionals and the institutions in which they work. The example of how compliment letters are investigated in research and handled by hospitals is an interesting indicator illustrating that the biopsychosocial model of care has not yet really taken root.

## Footnotes

1 We also conducted a comparative study in four other hospital settings – geriatric medicine, internal medicine, emergency medicine, and plastic and reconstructive surgery – and found similar results (short article in preparation).

## References

[ref1] Aparicio M, Centeno C, Carrasco JM, et al. (2017) What are families most grateful for after receiving palliative care? Content analysis of written documents received: A chance to improve the quality of care. *BMC Palliative Care* 16(1), 47. doi:10.1186/s12904-017-0229-5. 28874150 PMC5586049

[ref2] Aparicio M, Centeno C, Juliá G, et al. (2022a) Gratitude from patients and relatives in palliative care—characteristics and impact: A national survey. *BMJ Supportive & Palliative Care* 12(e4), e562–e569. doi:10.1136/bmjspcare-2019-001858. 31471493

[ref3] Aparicio M, Centeno C, Robinson C, et al. (2019) Gratitude between patients and their families and health professionals: A scoping review. *Journal of Nursing Management* 27(2), 286–300. doi:10.1111/jonm.12670. 30084234

[ref4] Aparicio M, Centeno C, Robinson CA, et al. (2022b) Palliative professionals’ experiences of receiving gratitude: A transformative and protective resource. *Qualitative Health Research* 32(7), 1126–1138. doi:10.1177/10497323221097247. 35574986 PMC9251753

[ref5] Balint M (1955) The doctor, his patient, and the illness. *The Lancet* 265(6866), 683–688. doi:10.1016/S0140-6736(55)91061-8. 14354967

[ref6] Bourquin C, Monti M, Saraga M, et al. (2022) Running against the clock: A qualitative study of internal medicine residents’ work experience. *Swiss Medical Weekly* 152, w30216. doi:10.4414/smw.2022.w30216. 36030393

[ref7] Clerc V, Stiefel F, Schaad B, et al. (2024) Beyond gratitude: A qualitative investigation of compliment letters received by a neonatology service, Submitted to BMC Health Services Research September 27 2024. 10.1186/s12913-026-14041-z PMC12911175 41580688

[ref8] Engel GL (1977) The need for a new medical model: A challenge for biomedicine. *Science* 196(4286), 129–136. doi:10.1126/science.847460. 847460

[ref9] Frank AW (1995) *The Wounded Storyteller: Body, Illness, and Ethics*. Chicago: University of Chicago Press.

[ref10] Gabbard GO (2017) *Long-Term Psychodynamic Psychotherapy: A Basic Text Third Edition*. Arlington: American Psychiatric Association Publishing.

[ref11] Gillespie A and Reader TW (2021) Identifying and encouraging high-quality healthcare: An analysis of the content and aims of patient letters of compliment. *BMJ Quality & Safety* 30(6), 484–492. doi:10.1136/bmjqs-2019-010077. PMC8142452 32641354

[ref12] Herbland A, Goldberg M, Garric N, et al. (2017) Thank you letters from patients in an intensive care unit: From the expression of gratitude to an applied ethic of care. *Intensive and Critical Care Nursing* 43, 47–54. doi:10.1016/j.iccn.2017.05.007. 28668642

[ref13] Khullar D (2024) The Gilded Age of Medicine Is Here. *The New Yorker*, 12 December. https://www.newyorker.com/culture/2024-in-review/the-gilded-age-of-medicine-is-here (accessed 11 March, 2025).

[ref14] Lloyd R, Munro J, Evans K, et al. (2023) Health service improvement using positive patient feedback: Systematic scoping review. *PLOS One* 18(10), e0275045. doi:10.1371/journal.pone.0275045. 37796785 PMC10553339

[ref15] Martins Pereira S and Hernández-Marrero P (2016) “In Memory of Those Who Left”: How “Thank You” letters are perceived and used as a team empowerment motivational factor by a home-based palliative care team in the Azorean Islands. *Journal of Palliative Medicine* 19(11), 1130–1131. doi:10.1089/jpm.2016.0255. 27327489

[ref16] Plunkett A (2022) Embracing excellence in healthcare: the role of positive feedback. *Archives of Disease in Childhood - Education and Practice* 107(5), 351–354. doi:10.1136/archdischild-2020-320882. 34426538

[ref17] Reader TW, Gillespie A and Roberts J (2014) Patient complaints in healthcare systems: A systematic review and coding taxonomy. *BMJ Quality & Safety* 23(8), 678–689. 10.1136/bmjqs-2013-002437. PMC4112446 24876289

[ref18] Schaad B, Bourquin C, Panese F, et al. (2019) How physicians make sense of their experience of being involved in hospital users’ complaints and the associated mediation. *BMC Health Services Research* 19(1), 73. doi:10.1186/s12913-019-3905-8. 30691452 PMC6348658

[ref19] Stirling FJ, Monteux S and Stoll M (2023) Receiving thank you letters in inpatient child and adolescent mental health services (CAMHS): A qualitative study of nurse’s experiences. *Journal of Psychiatric and Mental Health Nursing* 30(4), 709–718. doi:10.1111/jpm.12899. 36650671

